# Factors Affecting Post-EVAR Imaging Surveillance: An Opportunity for Improvement †

**DOI:** 10.3390/jcm15010039

**Published:** 2025-12-20

**Authors:** Daniel Gage, Drayson B. Campbell, Michael R. Go, Xiaoyi Teng, Kristine Orion

**Affiliations:** 1 Department of General Surgery, The Ohio State University, Columbus, OH 43210, USA; 2 Department of General Surgery, Division of Vascular Surgery, The Ohio State University, Columbus, OH 43210, USA; drayson.campbell@osumc.edu (D.B.C.); michael.go@osumc.edu (M.R.G.); xiaoyi.teng@osumc.edu (X.T.); kristine.orion@osumc.edu (K.O.)

**Keywords:** EVAR, aortic surgery, surveillance, follow-up

## Abstract

**Background/Objectives**: Appropriate imaging surveillance, established by the Society of Vascular Surgery (SVS), following endovascular aorta repair (EVAR) is critical for patient monitoring. We hypothesized that adherence to follow-up decreases over time, and therefore, the ability to detect endoleaks after EVAR also decreases. **Methods**: A retrospective cohort study of patients who underwent EVAR from 2014 to 2022 at our institution was completed. Patients were stratified by adherence to SVS guidelines for up to five years postoperatively. Demographics, detection of an endoleak > 30 days postoperatively, distance from our facility, and Area Deprivation Index (ADI) were collected. Comparisons of baseline comorbidities between groups and multivariate logistic regressions were performed using R studio. **Results**: 395 patients underwent an index EVAR at our institution from 2014–2022. 174 (44%) of patients adhered to all imaging recommendations, with an average loss to follow-up of 9.7% per year. 61 (15.4%) patients had a detected type II endoleak during the study period. Multivariable analysis identified residence > 50 miles from our institution as an independent risk factor for nonadherence (OR 1.76, *p* = 0.018) when controlling for age, sex, race, and ADI quartile. **Conclusions**: Adherence to surveillance guidelines gradually decreases after EVAR, but type II endoleak detection continues to occur years following the operation. While residence greater than 50 miles away was associated with nonadherence, patients’ ADI was not. Our results identify an opportunity for providers who may see patients more frequently to assist in reminding and arranging imaging follow-up for patients following their procedure.

## 1. Introduction

Endovascular aneurysm repair (EVAR) as a surgical intervention for abdominal aortic aneurysms (AAA) has increased greatly over the past few decades, offering a more minimally invasive approach compared to traditional open aortic aneurysm repair. In 2021, greater than 80% of all aortic aneurysm repairs were performed with EVAR, representing a vast majority of all cases [1].

Postoperative imaging surveillance after EVAR is vital to monitor potential complications, and the Society for Vascular Surgery (SVS) Guidelines recommend lifelong imaging surveillance following this procedure. Computed tomography angiography (CTA) is recommended for all patients for the first time one-month postoperatively to monitor for complications such as migration or endoleak, a process by which blood continues filling the aneurysm sac around the EVAR [2]. Endoleaks are common and can occur in up to 30% of patients following this procedure. Specifically, type II endoleaks are the most common, representing about 50% of all endoleaks and are usually due to continued filling of the aneurysm sac via retrograde flow from the stent-covered lumbar arteries or inferior mesenteric artery (IMA). While 90% of these endoleaks will spontaneously resolve, 10% will require additional surgical intervention [3]. If unmonitored, aortic aneurysm sac enlargement may occur and increase the risk of rupture. This underscores the importance of continued follow-up given the need for avoidance of potentially life-threatening complications. Monitoring by vascular surgeons alone is often suboptimal as patients are frequently lost to follow up, and primary care providers may function as a vital safety net for many patients. Vascular surgeons tend to lead follow up after surgery, but patients who see nonvascular surgery specialists have been shown to have lower rates of loss to vascular surgery follow up as well as a decreased five-year mortality [4].

We hypothesized that imaging adherence following EVAR decreases over time and would lead to a decrease in endoleak detection due to a failure in diagnosis. The purpose of our study was to describe the imaging surveillance adherence following EVAR at our large academic medical center, evaluate potential contributory patient-level factors, and examine type II endoleak detection over time.

## 2. Methods

### 2.1. Institutional Information

After approval from an Institutional Review Board (The Ohio State University Biomedical Sciences Institutional Review Board protocol number 2021H0268), we conducted a retrospective cohort study of patients with infrarenal AAA who received index EVAR at our institution from 2014–2022.

### 2.2. Patient Characteristics

The medical records of adult, non-inmate patients who received index EVAR at our institution from 2014–2022 were reviewed to collect baseline characteristics including age, sex, race, Hispanic/Latino ethnicity, and comorbidities including coronary artery disease, congestive heart failure, chronic obstructive pulmonary disease, diabetes, dialysis dependence, hypertension, smoking status, and chronic kidney disease.

Socioeconomic data collected included primary insurer and Area Deprivation Index (ADI). The ADI is a validated index that ranks neighborhoods (Census block groups) in the United States based on factors for the domains of income, education, employment, and housing quality; a higher score indicates worse deprivation [5,6]. ADI was split into four evenly sized quartiles. Utilizing patient addresses, the distance to our main medical campus was calculated. Distance was stratified into patients residing greater than 50 miles from the main medical center or less than 50 miles from the main medical center, in line with the mean distance and representing approximately a 1-h drive away.

### 2.3. Outcomes

Primary outcomes included survival, endoleak detection, endoleak intervention, and post-operative (post-op) imaging surveillance adherence. Endoleaks that were identified on completion angiogram at the index case but not seen at the next surveillance scan were excluded. Adherence to surveillance guidelines set by the Society for Vascular Surgery includes a one-month post-operative computed tomography angiogram (CTA), a six-month post-operative CTA if endoleak was present on the one-month scan, and annual CTA or ultrasound thereafter [2,7]. Patients’ adherence to indicated surveillance was tracked up to 5 years following the index surgical intervention unless they expired during this time. At our institution, patients frequently receive CT imaging for additional indications unrelated to their EVAR that are close to designated follow-up times, and our team will sometimes utilize this imaging to meet the surveillance recommendation to minimize additional contrast and radiation exposure. To account for more real-world follow-up patterns, patients were considered adherent at each time point if they received the indicated imaging in a window around the time point (one month ± one week, six months ± two months, annual scans ± two months). Additionally, patients were not required to have imaging completed with our institution; imaging available through Epic’s Care Everywhere or brought into the office at their visit (e.g., via compact disc, CD) was accepted. Patients were considered adherent if they received all recommended images and non-adherent if they missed any. For each time point, only applicable patients who underwent their index procedure in the appropriate time frame were considered.

### 2.4. Analysis

Two-sample independent *t*-tests were used to compare continuous variables (shown as mean ± standard deviation). Chi-squared or Fisher’s exact tests were used for categorical variables (shown as category counts and percentages). Univariable logistic regression was used to determine associations between patient characteristics and adherence to screening guidelines. Variables with a *p*-value of <0.2, along with preselected variables (age, sex, and non-white race), were included in a multivariable logistic regression to evaluate for independent associations. A geographical display of patient nonadherence was generated by determining county-level nonadherence rates and projecting these rates onto the map. All analyses were performed at an alpha level of 0.05. Analyses and figures were conducted using R Studio (Version 4.2.2).

## 3. Results

**Figure 1:**
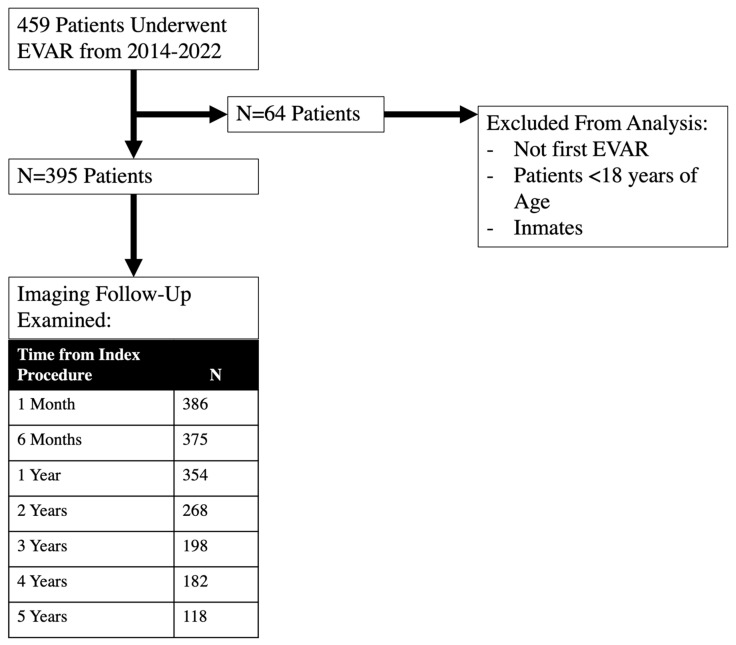
A description of the selection criteria for this study.

Our study included 395 patients who underwent EVAR (Figure 1). There was no difference in the baseline comorbidities between patients who were adherent with imaging follow up and those who were not. 82% of the patients in this study were male, but there was no difference in sex distribution between the two study groups. Patients who were non-adherent to imaging follow-up guidelines lived 47.2 miles from campus, while patients who were adherent lived 39.9 miles away (*p* = 0.024). 48.2% of patients who were non-adherent lived greater than 50 miles away from campus compared to 34.7% who were adherent (*p* = 0.010) (Table 1). Patients who were not adherent with imaging had worse ADI on univariate analysis (*p* = 0.014, Table 2), but this did not reach statistical significance on multivariate analysis (*p* = 0.181) (Table 3).

**Figure 2:**
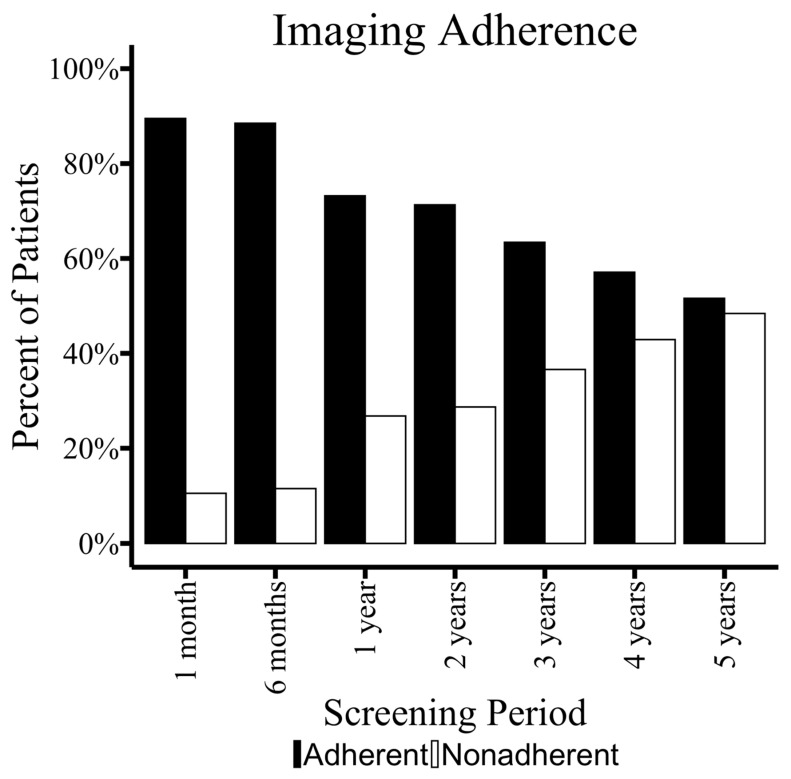
The percentages of patients adherent to the indicated screening time points are shown.

**Figure 3:**
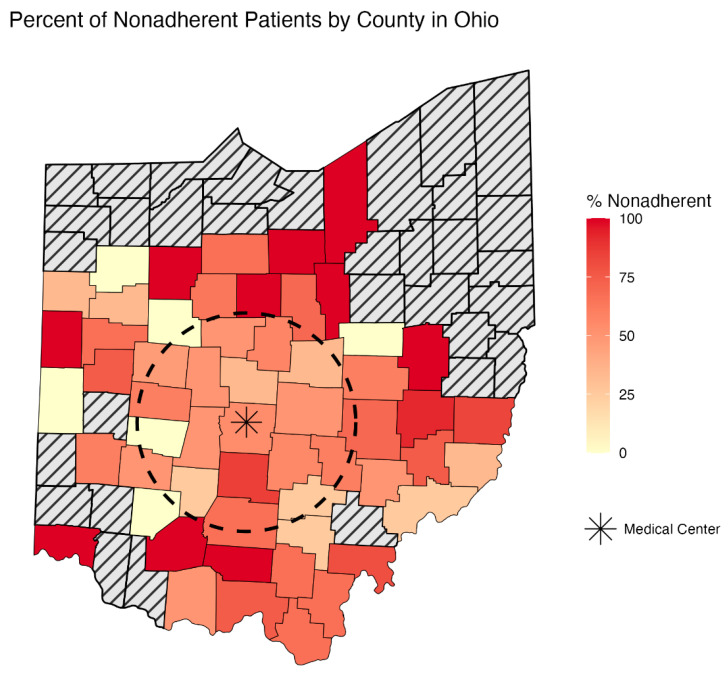
Patients living greater than 50 miles from our medical campus (dotted circle) had an increased risk of nonadherence with imaging follow-up recommendations.

**Figure 4:**
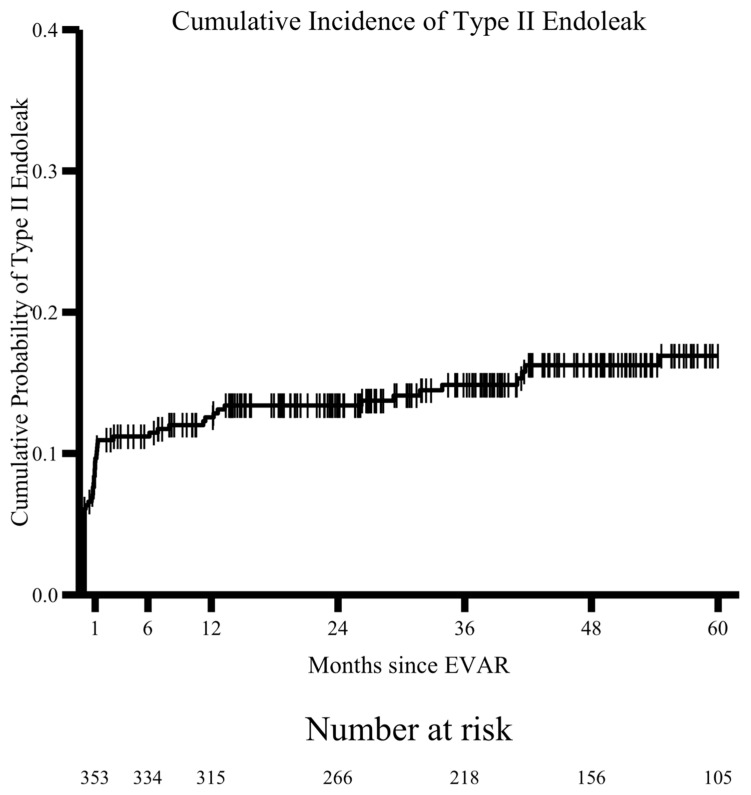
A Kaplan–Meier analysis depicts the cumulative incidence of type II endoleak over time.

When stratifying imaging follow-up among this cohort over 5 years postoperatively, 174 (44%) of patients adhered to all imaging guidelines. An average of an additional 9.7% of patients were deemed non-adherent each year of our study (Figure 2). ADI quartiles, residence >50 miles from the medical center, and the preselected variables age, sex, and race, in line with previous studies examining disparities in vascular surgery [8,9], were included in the multivariable analysis. This analysis identified residence > 50 miles as the only independent risk factor for non-adherence (OR 1.76, *p* = 0.018) (Table 3, Figure 3). Type II endoleaks were detected in 61 patients (15.4%) and at all time points assessed in this analysis (Table 1, Figure 4).

## 4. Discussion

The results from this study showed that imaging surveillance adherence following EVAR is poor overall, and the only patient-related factor associated with this adherence was distance from our medical campus. Continued imaging surveillance is vital to assess the success of aortic repair following EVAR and to determine the need for additional interventions. In-person follow-up alone following this procedure has shown a five-year survival benefit when compared to patients who were lost to follow-up as well as those with phone call follow-up [10].

Given the frequency of imaging follow-up required, previous studies have discussed concerns with adherence to these imaging guidelines [11,12]. The need for lifelong, frequent follow-up can often be confusing for patients undergoing the procedure, and interventions like the creation of education materials have helped in maintaining higher follow-up rates [13]. An examination of Medicare data demonstrated that 22% of patients are lost to follow up 1 year after EVAR and 50% are lost to follow up 5 years after EVAR. Within this analysis, increasing age, urgency of repair, and aneurysm rupture were associated with worse follow-up rates [9]. Additional analysis of data from Veteran’s Affairs (VA) hospitals showed similar rates of loss to follow up and associated patient history of smoking and alcohol use as well as several comorbidities with this observation [14]. Recent analyses showed late complications associated with EVAR, family history of AAA, and lack of social work referral, which the authors described as reflective of socioeconomic stability, were associated with higher compliance [15].

**Figure 5:**
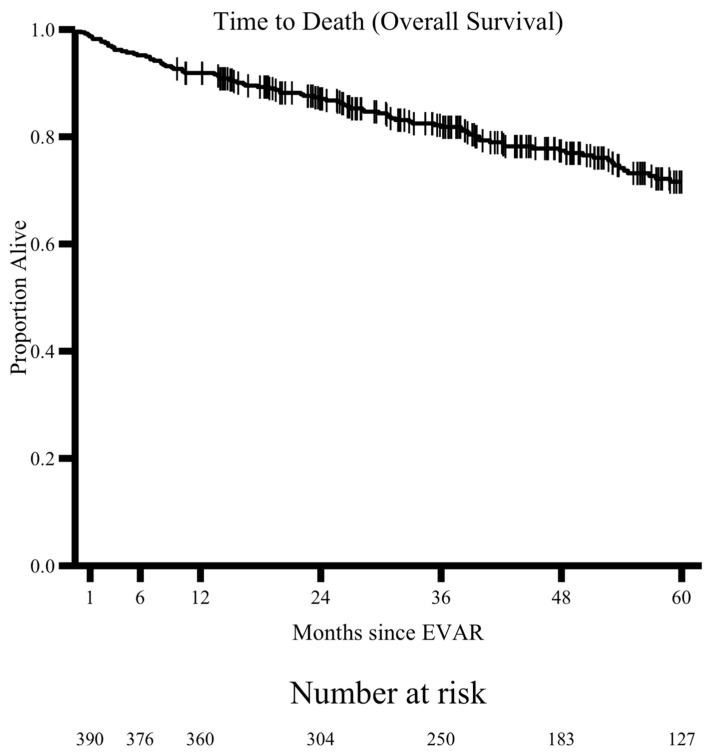
A Kaplan–Meier analysis showing the all-cause mortality of our cohort over time.

Our results suggest similar rates of loss to follow up over the first 5 years, with an average loss to follow up of 9.7% each year and approximately 50% adherence to guidelines at the end of this period. Over this time, 15.4% of patients in our cohort had type II endoleaks that were detected at least one month following their repair. They were detected at the highest rate at the time of the operation but continued throughout the five-year study period (Figure 4). A Kaplan–Meier Analysis examining all-cause mortality shows a steady rate during the study period, and univariate analysis demonstrated no difference in mortality between patients who were adherent to imaging guidelines and those who were not (Figure 5). This is in line with previous studies describing mortality rates following this procedure [16].

Our study allowed for the use of CTA or ultrasound at the appropriate time following the index operation, in line with current SVS recommendations, to determine adherence. Given differences in sensitivities in these methods, there may be more endoleaks which went undetected in this study, but detection of these endoleaks multiple years after the index operation underscores the importance of continued surveillance. Additionally, patients were given a window of time at each SVS-recommended imaging screening time in which they would still be deemed adherent to imaging guidelines by this analysis. While this may help account for more real-world imaging follow-up patterns, this may overestimate imaging adherence that may be present with more strict definitions of follow-up.

Further multivariate analysis of our cohort using the predictive variables age, sex, race, ADI quartile, and a home distance greater than 50 miles from our medical campus suggested that this distance was the only factor associated with a decreased rate of imaging follow-up. Adjusting for ADI quartile trended towards significance but did not demonstrate any differences between groups, suggesting that potential differences in access to care based on a patient’s neighborhood deprivation did not impact their ability to obtain appropriate imaging. This trend, however, may also suggest that the cohorts may be underpowered.

During our review, we considered patients adherent to recommendations if they received imaging at an outside facility, reflecting modern healthcare’s ability to share medical records electronically. Typically, these images are available for review at our facility even if obtained in a different healthcare system. Nonetheless, we still noted poor overall imaging follow-up in our cohort. Additional variables which we hypothesized may be important to a patient’s support system as well as ability to follow up, including marriage and insurance status, were examined, but group differences were not large enough for inclusion in multivariate analysis.

Primary care providers (PCPs) are vital touchstone in identifying patients with AAA, and screening has shown a decrease in AAA-related mortality or AAA rupture [17]. We believe this study, though limited, may offer a role to the impact that PCPs have in the post operative surveillance following EVAR. Studies have shown the impact of PCPs in early postoperative phase in decreasing rates of readmission following vascular surgery procedures [18,19]. In 2023, 79.4% of patients over the age of 18 reported having a wellness visit with a provider [20], and patients aged 65 years or older, an important population in patients undergoing EVAR, accounted for 42.5% of all primary care visits in the United States from 2018–2019 [21]. Additionally, Medicare beneficiaries averaged about 3 visits with a primary care provider per year in 2019 [22]. Like many chronic illnesses, EVAR requires long-term monitoring to ensure the best chances of a successful repair. Although their specific involvement was not captured in this analysis, the frequency of visits to PCPs by patients who may have had EVAR may be good opportunities to remind or assist patients to coordinate follow-up with their vascular surgeon.

Our data suggest there is significant room for improvement in adherence to imaging follow-up following EVAR and that continued interventions can be made to address potential barriers to care such as distance to the medical center. Further efforts should be made to work with patients who live farther from our facility to develop plans for access to these resources. We suspect that additional assistance from PCPs in reminding patients of the continued need for imaging represents a potential intervention to improve follow-up adherence and is an area for future study. Undetected endoleaks remain potentially life-threatening complications from this procedure and may become increasingly prevalent as more patients undergo the procedure for repair of their abdominal aortic aneurysms.

## 5. Conclusions

EVAR is an increasingly common surgical approach for the management of aortic aneurysms, and imaging follow-up is vital to the detection of endoleaks multiple years after the index operation. Adherence to these recommendations remains poor, with distance from the medical campus being a predictor of patients’ ability to obtain appropriate imaging. Incorporating imaging obtained outside of our facility still shows a low overall cohort follow-up rate, and additional efforts by patients’ care teams need to be made to reach patients to coordinate these studies.

## Figures and Tables

**Table 1:** Comparison of study groups.

Variable	Adherent N = 174	Nonadherent N = 221	*p*-Value
Age (years)	71.8 ± 8.0	70.6 ± 8.3	0.157
Male Sex	144 (82.8%)	181 (81.9%)	0.929
Non-white race	10 (5.8%)	14 (6.3%)	0.975
Married	131 (75.3%)	160 (72.4%)	0.595
Distance from medical center (miles)	39.9 ± 31.5	47.2 ± 32.0	0.024 *
Reside > 50 miles from medical center	60 (34.7%)	106 (48.2%)	0.010 *
ADI			0.053
1^st^ quartile (least deprived)	20 (12.9%)	10 (5.2%)	
2^nd^ quartile	30 (19.4%)	34 (17.5%)	
3^rd^ quartile	59 (38.1%)	78 (40.2%)	
4^th^ quartile (most deprived)	46 (29.7%)	72 (37.1%)	
Insurance			0.570
Commercial	49 (28.2%)	73 (33.0%)	
Government-assisted	122 (70.1%)	145 (65.6%)	
Self-pay	3 (1.7%)	3 (1.4%)	
CHF	15 (8.6%)	23 (10.4%)	0.670
COPD	67 (38.5%)	86 (38.9%)	1.000
CKD3b+	13 (7.5%)	30 (13.6%)	0.077
DM	38 (21.8%)	47 (21.3%)	0.989
HTN	149 (85.6%)	191 (86.4%)	0.937
Ever smoker	158 (90.8%)	197 (89.1%)	0.707
Endoleak †	59 (33.9%)	68 (30.8%)	0.579
Type I Endoleak	4 (2.3%)	12 (5.4%)	0.131
Type II Endoleak	34 (19.5%)	27 (12.2%)	0.063
Type III Endoleak	0 (0%)	3 (1.4%)	0.259
Reintervention	13 (7.5%)	15 (6.8%)	0.948
Death	37 (21.3%)	54 (24.4%)	0.534

Definitions: CHF (Congestive Heart Failure), COPD (Chronic Obstructive Pulmonary Disease), CKD3b+ (Chronic Kidney Disease, Stage IIIb or greater), DM (Diabetes Mellitus, unspecified whether Type I or Type II), HTN (Hypertension); * Significant *p* < 0.05; † Endoleaks without a clear source (i.e., those without a type specified in the CTA final read or in the vascular surgery clinic) are included.

**Table 2:** A univariate analysis comparing study groups. OR are reflective of OR if the variable answer is “yes”.

Characteristic	Univariable OR (95% CI)	*p* Value
Age	0.98 (0.96–1.01)	0.159
Female Sex	1.06 (0.63–1.79)	0.825
Nonwhite race	1.11 (0.48–2.56)	0.808
Married	0.86 (0.55–1.36)	0.518
ADI quartiles	1.33 (1.06–1.68)	0.014 ß,*
Noncommercial insurance	0.80 (0.53–1.21)	0.290
CHF	1.23 (0.62–2.44)	0.551
COPD	1.02 (0.68–1.53)	0.934
CKD 3b+	1.04 (0.85–1.27)	0.701
DM	0.97 (0.60–1.57)	0.891
HTN	1.07 (0.60–1.89)	0.821
Smoking	0.83 (0.43–1.62)	0.587
Reside > 50 miles from medical center	1.75 (1.16–2.64)	0.007 ß,*

ß included in multivariable analysis (Table 3) due to univariable *p* < 0.2; * Significant *p* < 0.05; Definitions: CHF (Congestive Heart Failure), COPD (Chronic Obstructive Pulmonary Disease), CKD 3b+ (Chronic Kidney Disease, Stage IIIb or greater), DM (Diabetes).

**Table 3:** Increasing OR means increasing risk of nonadherence. OR values are reflective of OR if the variable answer is “yes”. Unadjusted McFadden pseudo-R^2^ = 0.027.

Characteristic	Multivariable OR (95% CI)	*p* Value	Variance Inflation Factor, VIF (95% CI)
Age †	0.99 (0.62–1.01)	0.307	1.02 (1.00–3.23)
Female Sex †	1.08 (0.60–1.92)	0.806	1.02 (1.00–5.03)
Nonwhite race †	0.792 (0.210–2.12)	0.643	1.05 (1.00–1.51)
ADI quartiles ß	1.19 (0.92–1.52)	0.181	1.15 (1.06–1.37)
Reside > 50 miles from medical center ß	1.76 (1.10–2.80)	0.018 *	1.13 (1.05–1.35)

† included in multivariable analysis a priori; ß included in multivariable analysis due to univariable *p* < 0.2 (Table 3); * Significant *p* < 0.05.

## Data Availability

The de-identified data presented in this study is available upon request in compliance with institutional privacy policies.
